# Zero Moment Line—Universal Stability Parameter for Multi-Contact Systems in Three Dimensions

**DOI:** 10.3390/s22155656

**Published:** 2022-07-28

**Authors:** Tilen Brecelj, Tadej Petrič

**Affiliations:** Department of Automatics, Biocybernetics and Robotics, Jožef Stefan Institute, Jamova Cesta 39, 1000 Ljubljana, Slovenia; tadej.petric@ijs.si

**Keywords:** humanoid robotics, system stability, zero moment point, support polygon, biomechanics

## Abstract

The widely used stability parameter, the zero moment point (ZMP), which is usually defined on the ground, is redefined, in this paper, in two different ways to acquire a more general form that allows its application to systems that are not supported only on the ground, and therefore, their support polygon does not extend only on the floor. This way it allows to determine the stability of humanoid and other floating-based robots that are interacting with the environment at arbitrary heights. In the first redefinition, the ZMP is represented as a line containing all possible ZMPs, called the zero moment line (ZML), while in the second redefinition, the ZMP is represented as the ZMP angle, i.e., the angle between the ZML and the vertical line, passing through the center of mass (COM) of the investigated system. The first redefinition is useful in situations when the external forces and their acting locations are known, while the second redefinition can be applied in situations when the COM of the system under study is known and can be tracked. The first redefinition of the ZMP is also applied to two different measurements performed with two force plates, two force sensors, and the Optitrack system. In the first measurement, a subject stands up from a bench and sits down while being pulled by its hands, while in the second measurement, two subjects stand still, hold on to two double handles, and lean backward. In both cases, the stability of the subjects involved in the measurements is investigated and discussed.

## 1. Introduction

Robotic stability is a major challenge when it comes to the motion control of robots on a floating basis, i.e., robots that are not fixed onto the surface they are positioned on and can therefore flip over their support mechanism and fall. However, at the same time, robotic stability is of crucial importance as it determines whether a robot can perform a particular task in the desired manner or not. Due to the great progress that the development of floating-based robotic systems has made in the last few decades, the field of robot stability has expanded considerably and has experienced great advancement. This is especially true in the field of humanoid robotics, where robots attempt to mimic human-like behavior and interact effectively with humans [1,2]. Because humanoid robots are relatively large in contrast to the size of their feet, which support them, even the simplest tasks, such as walking or squatting, can be very challenging [3,4,5]. However, regardless of all these obstacles, the advancing research in robotics makes it possible to extend modern locomotion capabilities beyond the limits so that robots can run, jump, and even ski [6,7,8,9,10,11].

A robot is capable of performing the desired motion only if it is dynamically balanced. In this case, the motion of the robotic system must be controlled by the external forces acting on it. In addition, the sum of all existing forces must act somewhere along its support polygon (SP). If this is not the case, the external forces cannot fully explain the robot’s motion, which means that its behavior is not under control. In such scenarios, the robot is out of balance and it usually tips over its support area, which is, in the case of a humanoid robot, determined by its feet. To ensure the stability of the robot and prevent it from rotating about its SP, the horizontal moments must be zero at the point where the sum of all external forces acts on the system. This point is therefore called the zero moment point (ZMP) and is one of the most commonly used parameters for determining system stability [12,13].

**Figure 1:**
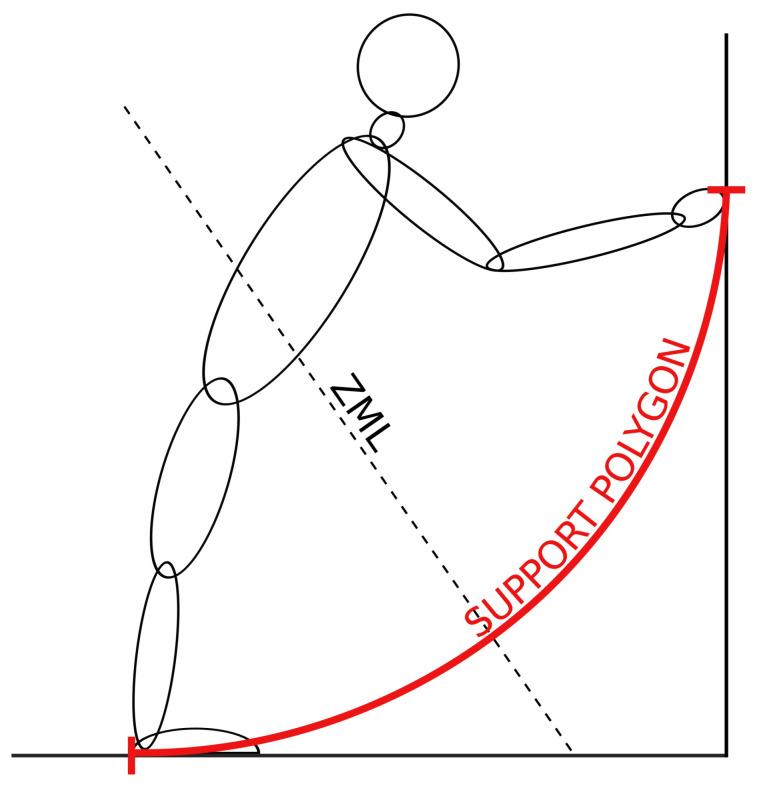
A humanoid, which can be either a human or a humanoid robot, supported by his feet on the ground and by his hands at a higher location. The SP, shown with the red curve, extends from the heel to the hands of the humanoid. The ZMP line passes through the humanoid and its SP, which indicates that the humanoid is stable.

According to its standard definition, the ZMP is located on the ground, which is useful in cases when the system is supported only on the ground and there are no external forces acting on the system at higher locations. Such scenarios have been thoroughly analyzed [14,15,16,17,18,19,20,21,22]. However, in situations that are very common in humanoid robotics, when the robot is supported at higher elevations, such as when it is sitting and supported by the bench or when it is either pushed or pulled by its hands, as shown in Figure 1, the standard definition of the ZMP on the ground cannot be used. Furthermore, if the center of mass (COM) of the robotic system is subjected to a large horizontal acceleration, the ZMP would be at a distance where the robot does not have suitable mechanisms to support itself. To overcome these limitations, several new methods have been developed. In most of the newly developed methods, the locations and the spatial and angular accelerations of the COM of each robot segment, along with their moments of inertia, must be known [23,24,25,26,27], while for some methods that also require the previously mentioned parameters, they are based on the creation of friction cones at the contacts of the robot with the environment [28,29,30,31,32,33,34,35,36,37,38,39,40].

However, in some situations, for example, in cases when the dimensions of the humanoid are not known, or when the locations and spatial and angular accelerations of its constituent parts, their COMs, their moments of inertia, and the friction coefficients and forces are not known, the existing methods for stability determination cannot be applied. This is especially true if the system under study is a human. In such situations, the ZMP can be redefined as a zero moment line (ZML), where the ZMP can be any point lying on this line [41,42], as depicted in Figure 1. The only parameters needed for the calculation of the ZML are the forces and torques acting on the investigated system along with their locations. This makes the ZML a universal parameter, which is simple to calculate and can be applied to any system, even if its dimensions, the locations, and the accelerations of its substituent parts are not known.

In this paper, the standard derivation of the ZMP is presented and extended to two more general forms, the ZML and the ZMP angle. The ZML is useful especially in situations when the external forces, torques, and their locations are known, as no other data are needed to obtain this stability parameter. On the other hand, the ZMP angle becomes the most convenient stability parameter to use in situations when the COM of the investigated system is known. These two newly defined parameters allow to monitor the stability of the investigated system in real time as well as determine the possible motions in accordance with the desired forces and their acting locations or the accelerations of the COM. This enables the motion planning for humanoid and other floating-based robots in a straightforward and simple way. In addition, in this paper, the SPs of a humanoid, which can be either a human or a robot, supported at different locations and with differently oriented forces, are illustrated and studied. Finally, the ZML is applied to the measurements of a subject sitting down on a bench and standing up while being pulled by its hands, as well as to the measurements of two subjects standing still, holding on to two double handles, and leaning backward. It is important to emphasize that the newly developed stability parameters, the ZML and the ZMP angle, were, in this paper and in [42], applied and tested on humans but can as well be applied to robotic or any other systems.

## 2. Methods

### 2.1. The Zero Moment Point

#### 2.1.1. The Standard Definition

With the standard definition, the stability parameter ZMP is defined as the point on the ground where the ground reaction force balances the entire system and prevents it from tipping and falling over its support mechanism. To stabilize the system, the ground reaction force must satisfy the torque balance equation, which states that the horizontal torques in the ZMP must be zero, and therefore, at this point, the system does not rotate about the horizontal axis.

**Figure 2:**
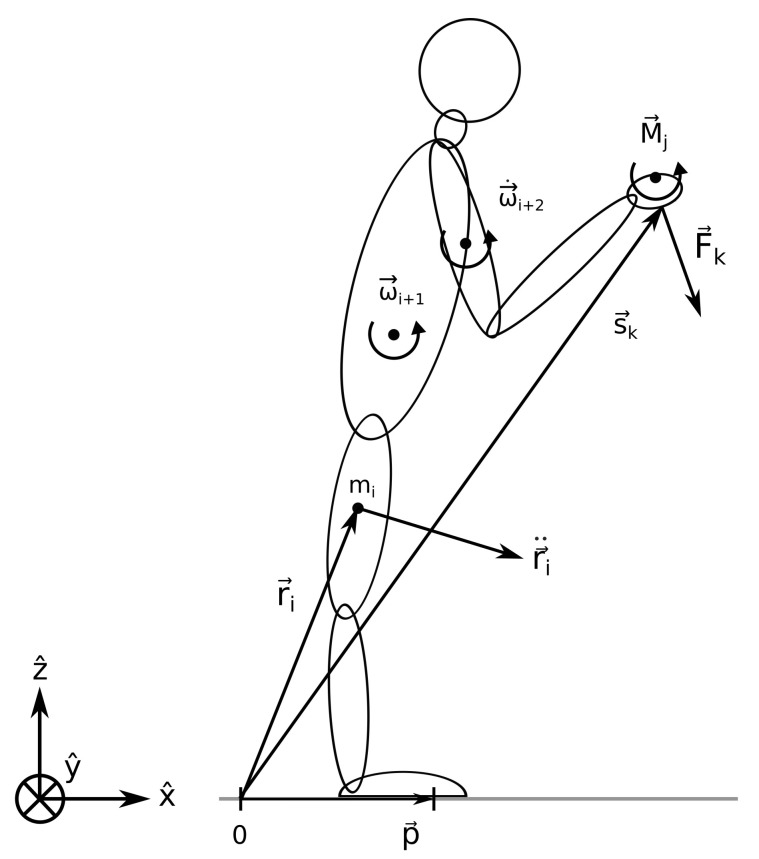
A humanoid with some exemplar external forces and torques. The orientation of the fixed coordinate system defining the space is shown in the lower left corner. See the text for the explanation of the symbols.

Let us consider a humanoid composed of multiple links, as shown in Figure 2.

The coordinate axes are aligned so that the sagittal plane of the humanoid is in the x−z plane, its lateral plane is in the y−z plane, and the ground plane is in the x−y plane. For such a system, the torque balance equation can be expressed as

(1) $$\sum_{i}\left[\left({\vec{r}}_{i}-\vec{p}\right)\times {\vec{F}}_{i}^{\mathrm{zmp}}+J_{i}{\dot{\vec{\omega }}}_{i}+{\vec{\omega }}_{i}\times J_{i}{\vec{\omega }}_{i}\right]-\sum_{j}{\vec{M}}_{j}-\sum_{k}\left({\vec{s}}_{k}-\vec{p}\right)\times {\vec{F}}_{k}={\vec{M}}_{\mathrm{zmp}}\;.$$

The location of the *i*-th link of the humanoid with respect to the origin of the coordinate system is pointed to by the vector ${\vec{r}}_{i}=\left(x_{i},y_{i},z_{i}\right)$; the ZMP is pointed to by the vector $\vec{p}=\left(p_{x},p_{y},p_{z}\right)$, with $p_{x}=p_{x}^{g}$ and $p_{z}=0$ when the ZMP lies on the ground; the ground reaction force acting from the ZMP to the *i*-th link is denoted by ${\vec{F}}_{i}^{\mathrm{zmp}}=m_{i}\left({\ddot{\vec{r}}}_{i}-\vec{g}\right)$, with $m_{i}$ being the mass of the *i*-th link; ${\ddot{\vec{r}}}_{i}=\left({\ddot{x}}_{i},{\ddot{y}}_{i},{\ddot{z}}_{i}\right)$ is its acceleration, while $\vec{g}=\left(0,0,g\right)$ is the vector of the gravitational acceleration and $g\approx -9.81\;m\;s^{-2}$; the moment of inertia matrix of the *i*-th link is $J_{i}=J_{\mu ,\lambda }^{i}$, where μ and λ are *x*, *y*, or *z*; the angular frequency and the angular acceleration of the *i*-th link are ${\vec{\omega }}_{i}$ and ${\dot{\vec{\omega }}}_{i}$, respectively; the *j*-th external torque is denoted by ${\vec{M}}_{j}=\left(M_{x}^{j},M_{y}^{j},M_{z}^{j}\right)$; the *k*-th external force is ${\vec{F}}_{k}=\left(F_{x}^{k},F_{y}^{k},F_{z}^{k}\right)$, acting at the location ${\vec{s}}_{k}=\left(x_{k},y_{k},z_{k}\right)$, while the torque at the ZMP is ${\vec{M}}_{\mathrm{zmp}}=\left(0,0,M_{z}^{\mathrm{zmp}}\right)$. The definition of the ZMP implies that the horizontal components of ${\vec{M}}_{\mathrm{zmp}}$ must be zero. On the other hand, its vertical component, $M_{z}^{\mathrm{zmp}}$, can in general be nonzero, as it is in normal circumstances balanced by the frictional forces [13]. An important thing to emphasize is that the force acting from the ZMP on the *i*-th link balances its gravitational attraction and accelerates it with the acceleration ${\ddot{\vec{r}}}_{i}$.

Some humanoid movements, such as, for example, sitting, standing, squatting, bending, etc., can be performed without rotations around the vertical axis. In such scenarios, there are only net forces acting along the sagittal plane at y=0, and therefore, there are only torques in the *y* direction. Consequently, the *y* component of the torque balance from Equation (1) becomes

(2) $$\begin{array}{cc} \; & \sum_{i}\left[m_{i}\left(z_{i}{\ddot{x}}_{i}-\left(x_{i}-p_{x}^{g}\right)\left({\ddot{z}}_{i}-g\right)\right)+J_{yy}^{i}{\dot{\omega }}_{y}^{i}\right]-\\ - & \sum_{j}M_{y}^{j}-\sum_{k}\left[z_{k}F_{x}^{k}-\left(x_{k}-p_{x}^{g}\right)F_{z}^{k}\right]=0\;. \end{array}$$

The *x* coordinate, where the ground reaction force that balances the humanoid acts, can therefore be expressed as

(3) $$p_{x}^{g}=\frac{\sum_{i}\left[m_{i}\left(x_{i}\left({\ddot{z}}_{i}-g\right)-z_{i}{\ddot{x}}_{i}\right)-J_{yy}^{i}{\dot{\omega }}_{y}^{i}\right]+\sum_{j}M_{y}^{j}+\sum_{k}\left[z_{k}F_{x}^{k}-x_{k}F_{z}^{k}\right]}{\sum_{i}m_{i}\left({\ddot{z}}_{i}-g\right)-\sum_{k}F_{z}^{k}}\;.$$

If this force lies within the SP, $p_{x}^{g}$ represents the location of the ZMP and coincides with the center of pressure (COP). In this case, the humanoid is in equilibrium and its motion is under control. However, if $p_{x}^{g}$ is outside the SP, the ZMP cannot exist because there are no mechanisms to support the humanoid outside the SP. In such situations, this location is called the fictitious ZMP [13] and the humanoid moves uncontrollably, usually leading to its fall.

When a humanoid is supported only by its feet, the SP lies beneath its feet and extends from the heel of the rear foot to the toes of the front foot and from the left edge of the left foot to the right edge of the right foot. When the feet are aligned with each other in the *x* direction, as shown in Figure 2, the SP extends in this direction from the heels to the toes.

A further simplification, in which only the ground reaction force acts on the humanoid and no torques and rotations are present, while the humanoid is approximated only by its COM, leads to the widely used model of the inverted pendulum [43]. In this approximation, the position of the ZMP from Equation (3) can be simplified to

(4) $$p_{x}^{g}=x_{\mathrm{com}}+\frac{z_{\mathrm{com}}}{g}{\ddot{x}}_{\mathrm{com}}$$

and it depends only on the horizontal and vertical coordinates of the COM, $x_{\mathrm{com}}$, and $z_{\mathrm{com}}$, respectively, and its horizontal acceleration, ${\ddot{x}}_{\mathrm{com}}$. To get a better insight into the inverted pendulum model, we rearrange Equation (4) into the form

$$\frac{x_{\mathrm{com}}-p_{x}^{g}}{z_{\mathrm{com}}}=-\frac{{\ddot{x}}_{\mathrm{com}}}{g}\;.$$

**Figure 3:**
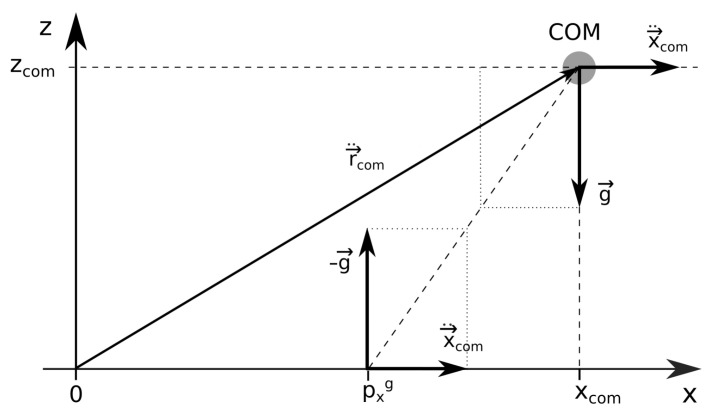
The linear inverted pendulum model with the accelerations of the COM and the accelerations produced by the ground reaction force acting at $p_{x}^{g}$.

The triangle defined by the COM and the ground locations of $x_{\mathrm{com}}$ and $p_{x}^{g}$ has the same ratio of the edges as the triangle, defined by the vectors of the COM acceleration in the *x* direction, ${\ddot{\vec{x}}}_{\mathrm{com}}=\left({\ddot{x}}_{\mathrm{com}},0,0\right)$, and the gravitational acceleration vector, as sketched in Figure 3.

The standard definition, where the ZMP is defined on the ground, is suitable mostly for cases when the system is supported solely on the ground, as it does not provide information on whether a system is stable if it is supported at higher altitudes. Furthermore, if the horizontal accelerations of the system are too large, the ZMP shifts to such far distances where the investigated system has no support mechanisms and the ZMP cannot be reached. Because of these limitations, more general definitions of the ZMP have been developed.

#### 2.1.2. The Line Definition

With the line definition, the ZMP can be anywhere on the ZML, including below the ground or above the investigated system. Furthermore, there are no restrictions on the COM acceleration as long as the system can support itself with sufficiently large forces at different heights, which is usually the case for humanoids.

If the system can be approximated with a single point with the mass $m_{\mathrm{com}}$, located at its COM, the sum of all the forces present can be expressed as

(5) $$m_{\mathrm{com}}\vec{g}+\sum_{j}{\vec{F}}_{j}=m_{\mathrm{com}}{\ddot{\vec{r}}}_{\mathrm{com}}\;,$$

where ${\vec{F}}_{j}=\left(F_{x}^{j},F_{y}^{j},F_{z}^{j}\right)$ is the *j*-th external force acting on the system and ${\ddot{\vec{r}}}_{\mathrm{com}}=\left({\ddot{r}}_{x}^{\mathrm{com}},{\ddot{r}}_{y}^{\mathrm{com}},{\ddot{r}}_{z}^{\mathrm{com}}\right)$ is the acceleration of its COM. It can be seen that the sum of all external forces balances the gravitational force and accelerates the COM with the acceleration ${\ddot{\vec{r}}}_{\mathrm{com}}$.

Assuming further that rotations of the system can be neglected and that any external force acts at the same point as its corresponding torque, the torque at any point *A* of the space, pointed by the vector $\vec{p}$, can be expressed as

(6) $${\vec{M}}_{A}=m_{\mathrm{com}}\left({\vec{r}}_{\mathrm{com}}-\vec{p}\right)\times \left(\vec{g}-{\ddot{\vec{r}}}_{\mathrm{com}}\right)+\sum_{j}[{\vec{M}}_{j}+\left({\vec{s}}_{j}-\vec{p}\right)\times {\vec{F}}_{j}]\;,$$

where ${\vec{r}}_{\mathrm{com}}$ and ${\vec{s}}_{j}=\left(s_{x}^{j},s_{y}^{j},s_{z}^{j}\right)$ point from the origin of the coordinate system to the COM and the *j*-th external force and torque, respectively. However, if point A lies in the ZMP, where the sum of all the torques is zero (assuming that the torque in the vertical direction is balanced by the friction forces and is therefore also zero), then ${\vec{M}}_{A}={\vec{M}}_{\mathrm{zmp}}=\left(0,0,0\right)$, and Equation (6) translates to

(7) $$m_{\mathrm{com}}\left({\vec{r}}_{\mathrm{com}}-\vec{p}\right)\times \left({\ddot{\vec{r}}}_{\mathrm{com}}-\vec{g}\right)=\sum_{j}[{\vec{M}}_{j}+\left({\vec{s}}_{j}-\vec{p}\right)\times {\vec{F}}_{j}]\;.$$

Equation (7) states that in the ZMP, the sum of all external torques acting on the system and the torques generated by the external forces acting on the system equals the torque generated by the force ${\vec{F}}_{\mathrm{zmp}}=m_{\mathrm{com}}\left({\ddot{\vec{r}}}_{\mathrm{com}}-\vec{g}\right)$ acting in the COM. Thus, all torques in the ZMP cancel each other and their sum is zero.

In practice, the precise location of the COM of the investigated system is rarely known, especially when the system has unknown exact dimensions and body-link configurations, such as a human. Therefore, the vector $\vec{p}$ pointing to the ZMP cannot be directly expressed from Equation (7). However, what is usually known are the external forces and torques acting on the system under study, as they can be easily measured. Thus, what can be calculated are the coordinates of the center of pressure on any horizontal plane at any height. The COP is defined as the location on a plane, where the sum of all the external forces should act, to produce the same torque, as it is produced on the same plane by all the external forces and torques.

**Figure 4:**
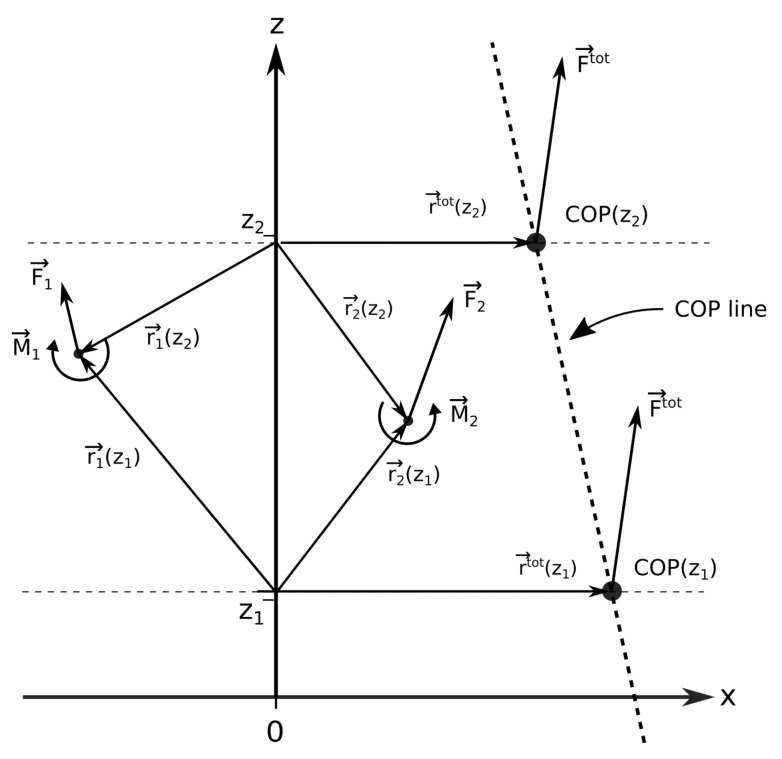
A schematic representation of two COPs on two horizontal planes at heights $z_{1}$ and $z_{2}$ and the COP line. ${\vec{F}}_{1}$ and ${\vec{F}}_{2}$ are two external forces, ${\vec{M}}_{1}$ and ${\vec{M}}_{2}$ are two external torques, the vectors ${\vec{r}}_{1}\left(z_{1}\right)$, ${\vec{r}}_{2}\left(z_{1}\right)$, ${\vec{r}}_{1}\left(z_{2}\right)$, and ${\vec{r}}_{2}\left(z_{2}\right)$ point from the centers of the two planes to the locations where the external forces and torques act, ${\vec{F}}^{\mathrm{tot}}$ is the sum of ${\vec{F}}_{1}$ and ${\vec{F}}_{2}$, while ${\vec{r}}^{\mathrm{cop}}\left(z_{1}\right)$ and ${\vec{r}}^{\mathrm{cop}}\left(z_{2}\right)$ are the vectors pointing from the centers of the two horizontal planes to their corresponding COP locations.

For the sake of simplicity, let us express the torque at the point Z=(x=0,y=0,z), lying on the vertical coordinate system axis at the height *z*, as

(8) $${\vec{M}}^{\mathrm{tot}}\left(z\right)={\vec{r}}^{\mathrm{cop}}\left(z\right)\times {\vec{F}}^{\mathrm{tot}}=\left(r_{y}^{\mathrm{cop}}F_{z}^{\mathrm{tot}}-r_{z}^{\mathrm{cop}}F_{y}^{\mathrm{tot}},r_{z}^{\mathrm{cop}}F_{x}^{\mathrm{tot}}-r_{x}^{\mathrm{cop}}F_{z}^{\mathrm{tot}},r_{x}^{\mathrm{cop}}F_{y}^{\mathrm{tot}}-r_{y}^{\mathrm{cop}}F_{x}^{\mathrm{tot}}\right)\;,$$

where

(9) $${\vec{M}}^{\mathrm{tot}}\left(z\right)=\left(M_{x}^{\mathrm{tot}}\left(z\right),M_{y}^{\mathrm{tot}}\left(z\right),M_{z}^{\mathrm{tot}}\left(z\right)\right)=\sum_{j}[{\vec{M}}_{j}+{\vec{r}}_{j}\left(z\right)\times {\vec{F}}_{j}]$$

is the total torque in the point *Z*, resulting from the external torques and forces acting on the investigated system, ${\vec{r}}_{j}\left(z\right)$ is the vector pointing from *Z* to the location where ${\vec{F}}_{j}$ and ${\vec{M}}_{j}$ act, ${\vec{r}}^{\mathrm{cop}}\left(z\right)=\left(r_{x}^{\mathrm{cop}}\left(z\right),r_{y}^{\mathrm{cop}}\left(z\right),r_{z}^{\mathrm{cop}}\left(z\right)\right)$ is the horizontal vector pointing from the point *Z* to the COP on the plane at the height *z*, and

(10) $${\vec{F}}^{\mathrm{tot}}=\left(F_{x}^{\mathrm{tot}},F_{y}^{\mathrm{tot}},F_{z}^{\mathrm{tot}}\right)=\sum_{j}{\vec{F}}_{j}\;,$$

as shown in Figure 4.

Because the vector ${\vec{r}}^{\mathrm{cop}}\left(z\right)$ lies on a horizontal plane, its vertical component equals zero, $r_{z}^{\mathrm{cop}}=0$, and is in Equation (8), written only for completeness. The *x* and *y* locations of the COP at a certain height *z* can therefore be calculated from Equation (8) as

(11) $$r_{x}^{\mathrm{cop}}\left(z\right)=-\frac{M_{y}^{\mathrm{tot}}\left(z\right)}{F_{z}^{\mathrm{tot}}}$$

and

(12) $$r_{y}^{\mathrm{cop}}\left(z\right)=\frac{M_{x}^{\mathrm{tot}}\left(z\right)}{F_{z}^{\mathrm{tot}}}\;.$$

Centers of pressure at different heights lie at different locations, but they are all on the same line, the COP line. One way to obtain the COP line is to determine the positions of two COPs at two different heights, $z_{1}$ and $z_{2}$, and calculate the parameters of the line that passes through them. Because, in this paper, the COP line is presented separately for the sagittal and lateral planes, the parameters of the COP lines lying in both planes are derived. The slopes of the COP lines lying in the x−z and y−z planes can be calculated as

(13) $$k_{xz}^{\mathrm{cop}}=\frac{z_{2}-z_{1}}{r_{x}^{\mathrm{cop}}\left(z_{2}\right)-r_{x}^{\mathrm{cop}}\left(z_{1}\right)}$$

and

(14) $$k_{yz}^{\mathrm{cop}}=\frac{z_{2}-z_{1}}{r_{y}^{\mathrm{cop}}\left(z_{2}\right)-r_{y}^{\mathrm{cop}}\left(z_{1}\right)}\;,$$

while their points of intersection with the vertical coordinate system axis can be calculated, either from the coordinates of the first COP at height $z_{1}$ or from the coordinates of the second COP at height $z_{2}$, such as

(15) $$n_{xz}^{\mathrm{cop}}=z_{1,2}-k_{xz}^{\mathrm{cop}}r_{x}^{\mathrm{cop}}\left(z_{1,2}\right)$$

and

(16) $$n_{yz}^{\mathrm{cop}}=z_{1,2}-k_{yz}^{\mathrm{cop}}r_{y}^{\mathrm{cop}}\left(z_{1,2}\right)\;.$$

Equations (13)–(16) can be, by the insertion of Equations (9)–(12), further simplified to

(17) $$k_{xz}^{\mathrm{cop}}=\frac{F_{z}^{\mathrm{tot}}}{F_{x}^{\mathrm{tot}}}\;,$$

(18) $$k_{yz}^{\mathrm{cop}}=\frac{F_{z}^{\mathrm{tot}}}{F_{y}^{\mathrm{tot}}}\;,$$

(19) $$n_{xz}^{\mathrm{cop}}=\frac{\sum_{j}(M_{y}^{j}+s_{z}^{j}F_{x}^{j}-r_{x}^{j}F_{z}^{j})}{F_{x}^{\mathrm{tot}}}$$

and

(20) $$n_{yz}^{\mathrm{cop}}=\frac{\sum_{j}(-M_{x}^{j}+s_{z}^{j}F_{y}^{j}-r_{y}^{j}F_{z}^{j})}{F_{y}^{\mathrm{tot}}}\;.$$

A detailed derivation of Equations (17) and (19) can be found in Appendix A. The derivations of Equations (18) and (20) can be performed in the same way as the derivations of Equations (17) and (19) and are therefore not included in this paper. As it can be seen from Equations (17)–(20), the parameters of the COP lines are independent of the heights $z_{1}$ and $z_{2}$, which further proves that all the COPs lie on the same line.

If Equation (7) holds and the system is stable, the sum of all existing torques in all COPs on any horizontal plane at any height is zero. In this case, all COPs are ZMPs and the COP line, corresponding to the ZML, passes through the SP of the investigated system.

#### 2.1.3. The Angular Definition

If the system can be approximated by its COM, which can be well determined and tracked over time, the angle definition of the ZMP can be used [41,42]. Given the same assumptions as in the derivation of Equation (2), except that the ZMP need not be on the ground in this case, the *y* component of the torque balance from Equation (1) becomes

(21) $$\begin{array}{cc} \; & \sum_{i}\left[m_{i}\left(\left(z_{i}-p_{z}\right){\ddot{x}}_{i}-\left(x_{i}-p_{x}\right)\left({\ddot{z}}_{i}-g\right)\right)+J_{yy}^{i}{\dot{\omega }}_{y}^{i}\right]-\\ - & \sum_{j}M_{y}^{j}-\sum_{k}\left[\left(z_{k}-p_{z}\right)F_{x}^{k}-\left(x_{k}-p_{x}\right)F_{z}^{k}\right]=0\;. \end{array}$$

Expressing the dependence of the *z* coordinate of the ZMP, $p_{z}$, as a function of its *x* coordinate, $p_{x}$, we obtain that all possible positions of the ZMP lie on the ZML, given by

(22) $$p_{z}=k_{\mathrm{zmp}}p_{x}+n_{\mathrm{zmp}}\;,$$

where

$$k_{\mathrm{zmp}}=\frac{\sum_{i}m_{i}\left({\ddot{z}}_{i}-g\right)-\sum_{k}F_{z}^{k}}{\sum_{i}m_{i}{\ddot{x}}_{i}-\sum_{k}F_{x}^{k}}$$

and

$$n_{\mathrm{zmp}}=\frac{\sum_{i}\left[m_{i}\left(x_{i}\left({\ddot{z}}_{i}-g\right)-z_{i}{\ddot{x}}_{i}\right)-J_{yy}^{i}{\dot{\omega }}_{y}^{i}\right]+\sum_{j}M_{y}^{j}+\sum_{k}\left[z_{k}F_{x}^{k}-x_{k}F_{z}^{k}\right]}{\sum_{k}F_{x}^{k}-\sum_{i}m_{i}{\ddot{x}}_{i}}\;.$$

**Figure 5:**
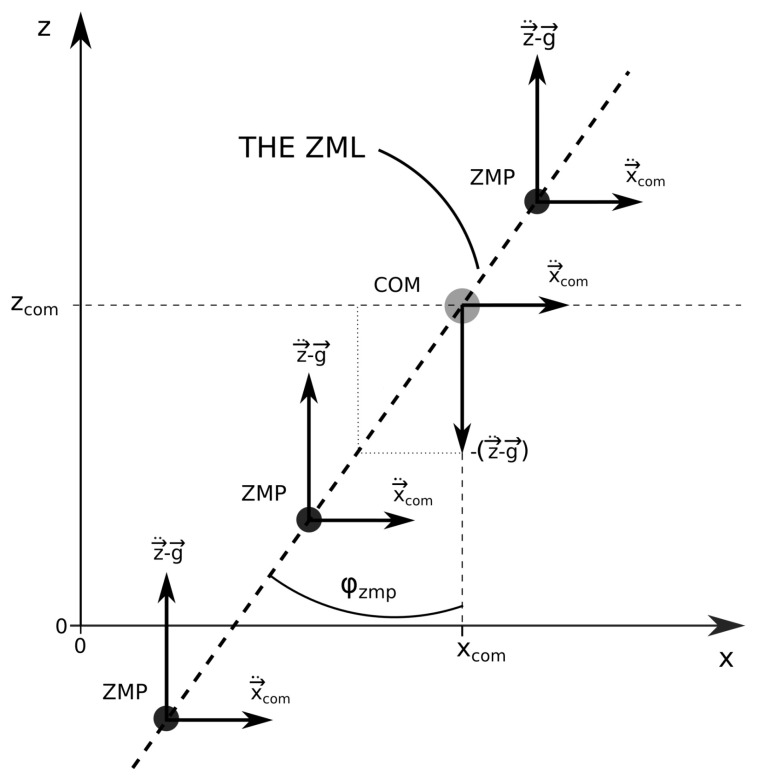
The ZML passing through the COM, with three different locations of the ZMP, such that $z_{\mathrm{zmp}}>z_{\mathrm{com}}$, $0<z_{\mathrm{zmp}}<z_{\mathrm{com}}$, and $z_{\mathrm{zmp}}<0$, respectively. $\varphi_{\mathrm{zmp}}$ is defined as the angle between the ZML and the vertical line, which is for a clearer representation passing through the COM.

If the rotations of the investigated system and the external torques present can be neglected and if the only external force acting on the system is the ground reaction force, the ZML defined by Equation (21) passes through the COM of the investigated system, as shown in Figure 5.

The angle between the ZML and the vertical line can now be defined as the ZMP angle, $\varphi_{\mathrm{zmp}}$, which can be expressed as

$$\varphi_{\mathrm{zmp}}=-\arctan2\left({\ddot{x}}_{\mathrm{com}},\left({\ddot{z}}_{\mathrm{com}}-g\right)\right)\;.$$

**Figure 6:**
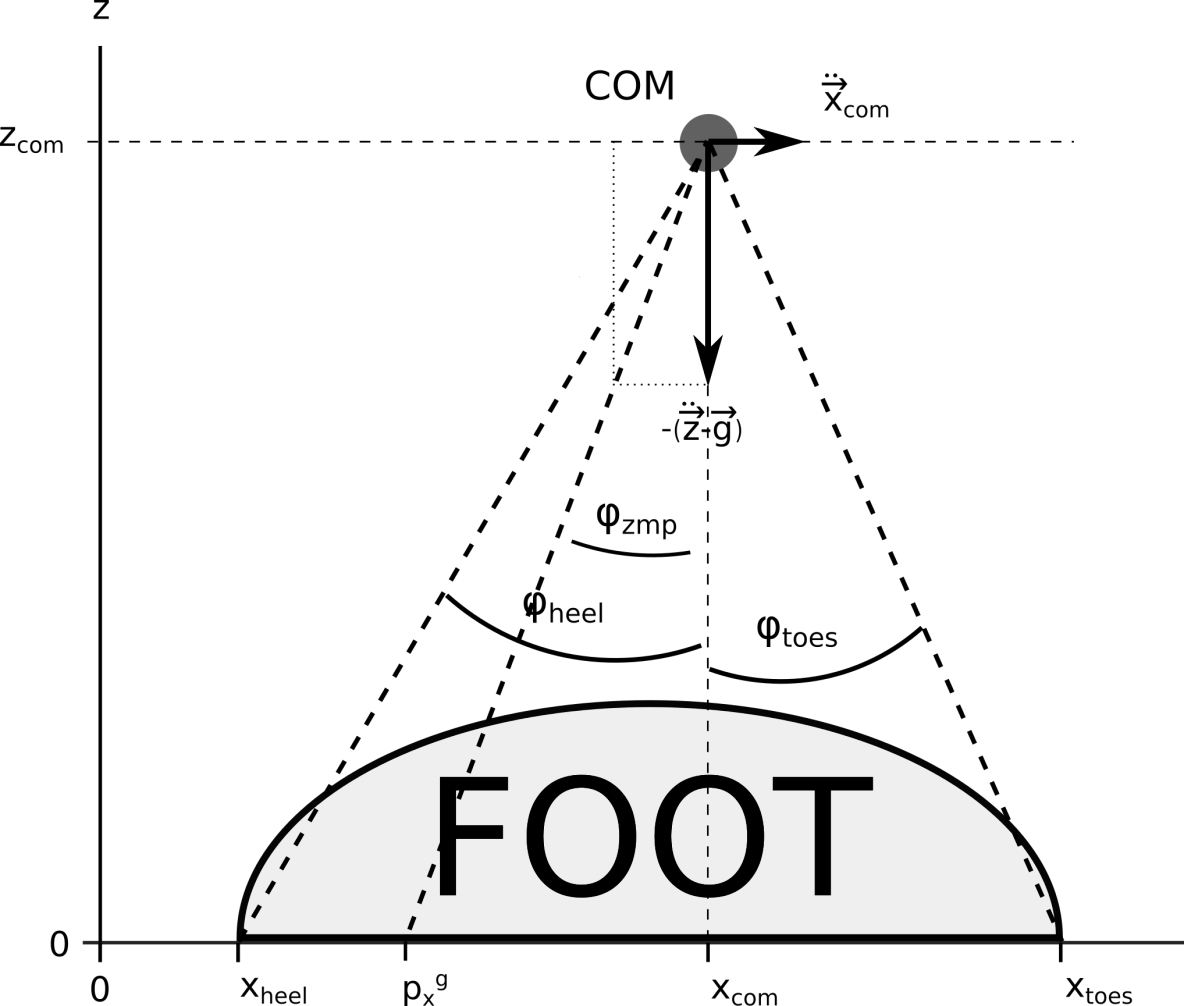
The ZMP angle and the coordinates, $x_{\mathrm{heel}}$ and $x_{\mathrm{toes}}$, and the angles, $\varphi_{\mathrm{heel}}$ and $\varphi_{\mathrm{toes}}$, of the edges of the SP, being the heel and the toes of the foot of the humanoid, with respect to its COM.

The support polygon can also be defined in the angular way. The angles of its edges are the angles between the lines, connecting the edges of the SP and the COM, and the vertical line, passing through the COM. As the angles of the SP of the investigated system are defined from its COM, the ZMP angle is for clarity and a direct and lucid comparison with the angles of the SP also presented as the angle around the vertical line passing through the COM of the investigated system. The angular definition of the ZMP and the SP of a humanoid, being supported only by its feet, is sketched in Figure 6.

If $\varphi_{\mathrm{zmp}}$ lies within the angles of the SP, the investigated system is stable, while if $\varphi_{\mathrm{zmp}}$ lies outside the angles of the SP, the system being observed is unstable.

A more detailed explanation of the angular definition of the ZMP can be found in [41], while its application can be found in [42].

### 2.2. Stability and the Support Polygon

The support polygon is the region around the investigated system, extending between the outermost points at which it can be supported. Thus, it is not necessarily a physical polygon anymore, as was the case when the system was supported only on the ground, but as the system can be supported at different heights, the SP now becomes a spatial region, extending between the outermost points where the support mechanisms are acting. However, the side of a system around which the SP extends depends on the orientation of the supporting forces. If a humanoid is sitting on a bench with its feet on the ground, the forces acting from the bench and the ground are oriented upward and can therefore be denoted as pushing forces. However, if someone helps the humanoid stand up or sit down by holding its hands, the forces can be either pushing or pulling.

**Figure 7:**
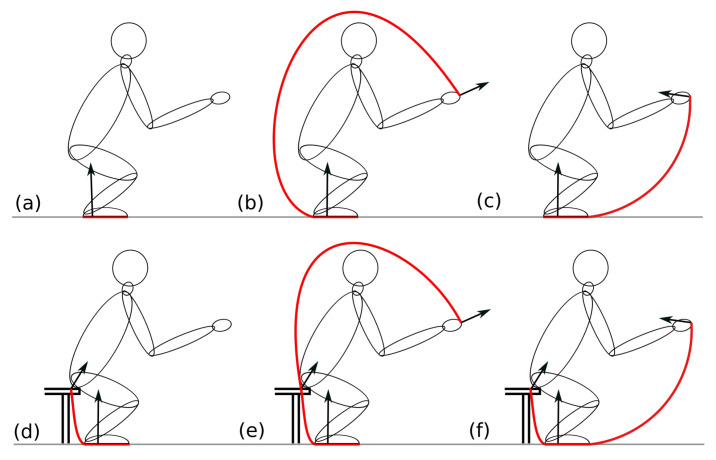
Support polygons (red curves) around a humanoid in its sagittal plane for different scenarios when the humanoid is standing up and is (**a**) supported only by its feet, (**b**) supported by its feet and pulled by its hands, (**c**) supported by its feet and pushed by its hands, (**d**) supported by its feet and its buttocks, (**e**) supported by its feet and by its buttocks while being pulled by its hands, and (**f**) supported by its feet and by its buttocks while being pushed by its hands. The black arrows represent the external forces acting on the humanoid.

Figure 7 shows the SPs around a humanoid for different scenarios, when it is standing up and is supported at different points, with forces oriented in different directions. In the case when the humanoid is supported only by its feet positioned on the ground, the SP extends between the outermost regions where its feet can touch the ground, as shown in Figure 7a. However, if the humanoid is also supported by the hands at a higher location, the SP extends between the furthest locations of its feet and its hands. If the forces acting on the hands are pulling forces, the SP extends from the hands around the back to the toes. However, when the forces acting on the hands are pushing forces, the SP extends in the opposite direction from the hands to the heels, which is shown in Figure 7b,c, respectively. When the humanoid is seated and is therefore supported both by its feet and its buttocks, the SP extends from its toes to the furthest point on the bench where support can still be granted, as shown in Figure 7d. Finally, when the humanoid is supported by its feet, by its buttocks, and by its hands, the SP encompasses the SPs shown in Figure 7b–d, as shown in Figure 7e,f, where the side around the humanoid on which the SP extends depends on whether the forces acting in the hands are pushing or pulling.

The condition that must be fulfilled for the humanoid to be stable is that the ZML must cross the humanoid and the SP, while ${\vec{F}}_{\mathrm{zmp}}$ points from the SP toward the humanoid. If this holds, the external forces acting on the humanoid can produce the torque needed to balance its gravitational force and accelerate its COM in the observed direction with the observed acceleration, as it is described by Equation (7). If this is not the case and the ZML does not cross the SP of the humanoid, the sum of the external forces cannot balance all the torques as it cannot act on the proper location and therefore stability cannot be provided. In the cases presented in Figure 7b–f, where the forces do not act only on the ground but also on higher locations, using the standard definition of the ZMP on the ground, the stability of the humanoid cannot be determined and this is why the redefinitions of the ZMP, derived in this paper, are needed.

## 3. Results—Application of the Line Definition

To evaluate the line definition of the ZMP, two different measurements of two interacting individuals were performed, and all the forces and their corresponding torques were recorded. The tasks performed in these measurements were chosen because they are easily applicable to humanoid robots and represent actions that humanoid robots can perform or are similar to those that they will perform when interacting with humans. At the same time, the chosen tasks include forces that are acting also above the ground and therefore allow the application and evaluation of the newly defined line definition of the ZMP. As the COMs of the individuals are not known, the ZML is the most suitable parameter for the determination of their stability. The analysis and the results of these two research cases are presented below.

### 3.1. Standing Up with Help

**Figure 8:**
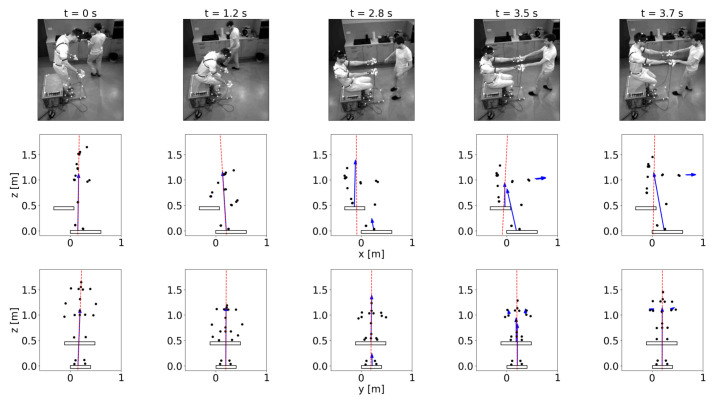
Outtakes of the recorded motion of a subject sitting down and standing up. The first row shows the photos of the measurement, while the second and the third rows show the sagittal and lateral planes, respectively. The photo and the figures from the same column correspond to the same time frame, noted at the top of the column. The bright dots positioned on the recorded subject, on the force plates, and the force sensors are the reflective markers of the Optitrack system. The black dots in the outtakes from the second and the third row represent the reconstructed joints of the recorded subject, the black rectangles represent the force plates positioned on the ground and the bench, respectively, the blue arrows emerging from the force plates represent the forces measured with the force plates, the blue arrows emerging from the hands represent the forces measured with the force sensors mounted on the double handles, and the red dotted line is the ZML, calculated as described in Section 2.1.2.

In this research case, the ZML was determined for a subject who sat down on a bench and stood up while it was pulled by its hands, as can be seen in the photo outtakes of the measurement presented in the first row of Figure 8.

In the first frame, the subject stands on his feet and is starting to sit down; in the second frame, he is about to sit on the bench; in the third frame, the subject is sitting on the bench with his legs on the floor; in the fourth frame, he is still sitting on the bench while being pulled by his hands; and in the last frame, the subject is standing up while being pulled by his hands.

To calculate the ZML, all external forces and torques acting on the subject were measured. The ground reaction force and the bench force were measured with two force plates [44] positioned on the ground and the bench, respectively, while the forces in the hands were measured with two force sensors [45] mounted on two double handles. On each double handle, the force sensor is attached between two handles, one for the subject standing up and one for the subject pulling.

The force plates used for the measurements discussed in this paper provide us with the measured forces and torques at the center of their top surface, while the force sensors provide us with the measured forces and torques at their own centers between the two handles. The positions of these measuring devices were tracked with the OptiTrack system [46], which detects the positions of the reflective markers that were placed on top of each measuring device. The reflective markers were also positioned on the subject that sat down and stood up, so that not only could its movements be tracked, but its skeleton could also be reconstructed using Motive software [47]. With the position of the skeleton, the reconstruction of the SP was finally possible.

The force balance can be, for the individual that sat down and stood up, expressed from Equation (5) as

(23) $$m_{\mathrm{com}}\left({\ddot{\vec{r}}}_{\mathrm{com}}-\vec{g}\right)={\vec{F}}_{f}+{\vec{F}}_{b}+{\vec{F}}_{\mathrm{lh}}+{\vec{F}}_{\mathrm{rh}}\;,$$

where ${\vec{F}}_{f}=\left(F_{x}^{f},F_{y}^{f},F_{z}^{f}\right)$ is the ground reaction force (f denotes the floor), ${\vec{F}}_{b}=\left(F_{x}^{b},F_{y}^{b},F_{z}^{b}\right)$ is the force acting from the bench (b denotes the bench) when the subject and the bench are in contact, while ${\vec{F}}_{\mathrm{lh}}=\left(F_{x}^{\mathrm{lh}},F_{y}^{\mathrm{lh}},F_{z}^{\mathrm{lh}}\right)$ and ${\vec{F}}_{\mathrm{rh}}=\left(F_{x}^{\mathrm{rh}},F_{y}^{\mathrm{rh}},F_{z}^{\mathrm{rh}}\right)$ are the forces acting on the left and the right hand of the subject that was standing up, respectively (lh and rh denote the left and the right hand, respectively).

Throughout the measurement, the subject that sat down and stood up was stable, because its movement was always under control and it did not tip over its SP. This implies that Equation (7) was satisfied throughout the measurement. Here, the ZML was computed from the measured forces from the right hand side of Equation (22) and their corresponding torques ${\vec{M}}_{f}=\left(M_{x}^{f},M_{y}^{f},M_{z}^{f}\right)$, ${\vec{M}}_{b}=\left(M_{x}^{b},M_{y}^{b},M_{z}^{b}\right)$, ${\vec{M}}_{\mathrm{lh}}=\left(M_{x}^{\mathrm{lh}},M_{y}^{\mathrm{lh}},M_{z}^{\mathrm{lh}}\right)$, and ${\vec{M}}_{\mathrm{rh}}=\left(M_{x}^{\mathrm{rh}},M_{y}^{\mathrm{rh}},M_{z}^{\mathrm{rh}}\right)$, respectively, as implied by the line definition presented in Section 2.1.2. The horizontal torque components in the center of a plane at height *z*, i.e., in the point (0, 0, *z*), can be expressed from Equation (9) as

$$M_{x}^{\mathrm{tot}}\left(z\right)=M_{x}^{f}+r_{y}^{f}F_{z}^{f}-r_{z}^{f}F_{y}^{f}+M_{x}^{b}+r_{y}^{b}F_{z}^{b}-r_{z}^{b}F_{y}^{b}+M_{x}^{\mathrm{lh}}+r_{y}^{\mathrm{lh}}F_{z}^{\mathrm{lh}}-r_{z}^{\mathrm{lh}}F_{y}^{\mathrm{lh}}+M_{x}^{\mathrm{rh}}+r_{y}^{\mathrm{rh}}F_{z}^{\mathrm{rh}}-r_{z}^{\mathrm{rh}}F_{y}^{\mathrm{rh}}$$

and

$$M_{y}^{\mathrm{tot}}\left(z\right)=M_{y}^{f}+r_{z}^{f}F_{x}^{f}-r_{x}^{f}F_{z}^{f}+M_{y}^{b}+r_{z}^{b}F_{x}^{b}-r_{x}^{b}F_{z}^{b}+M_{y}^{\mathrm{lh}}+r_{z}^{\mathrm{lh}}F_{x}^{\mathrm{lh}}-r_{x}^{\mathrm{lh}}F_{z}^{\mathrm{lh}}+M_{y}^{\mathrm{rh}}+r_{z}^{\mathrm{rh}}F_{x}^{\mathrm{rh}}-r_{x}^{\mathrm{rh}}F_{z}^{\mathrm{rh}}\;,$$

where ${\vec{r}}_{f}\left(z\right)=\left(r_{x}^{f},r_{y}^{f},r_{z}^{f}\left(z\right)\right)$, ${\vec{r}}_{b}\left(z\right)=\left(r_{x}^{b},r_{y}^{b},r_{z}^{b}\left(z\right)\right)$, ${\vec{r}}_{\mathrm{lh}}\left(z\right)=\left(r_{x}^{\mathrm{lh}},r_{y}^{\mathrm{lh}},r_{z}^{\mathrm{lh}}\left(z\right)\right)$, and ${\vec{r}}_{\mathrm{rh}}\left(z\right)=\left(r_{x}^{\mathrm{rh}},r_{y}^{\mathrm{rh}},r_{z}^{\mathrm{rh}}\left(z\right)\right)$ are vectors pointing from the point (0, 0, *z*) to the location where their corresponding forces and torques act. Having $M_{x}^{\mathrm{tot}}\left(z\right)$, $M_{y}^{\mathrm{tot}}\left(z\right)$ and the total vertical force

$$F_{z}^{\mathrm{tot}}=F_{z}^{f}+F_{z}^{b}+F_{z}^{\mathrm{lh}}+F_{z}^{\mathrm{rh}}\;,$$

obtained from Equation (10), the horizontal coordinates of COP, which coincide with the ZMP because the system is balanced, can be obtained from Equations (11) and (12). To obtain the ZML, the location of the COP at two different heights must be calculated. The coefficient of the ZML and its intersection with the vertical axis can then be obtained from Equations (13)–(16) or (17)–(20) for both the sagittal and lateral planes.

As in the first and the second frame, the subject is supported only by the force plate positioned on the ground, and the only external force acting is the reaction force exerted by the force plate. Because the subject is supported only by its feet and is stable, the ZML passes through the SP between its feet, as shown in Figure 7a. In the third frame, there are two external forces acting on the subject, one from the bench force plate and the other from the floor force plate, as it is sitting on the bench while being supported by its legs on the floor. In this case, the SP extends from its toes to its buttocks, as shown in Figure 7d. In the fourth frame, in addition to the forces acting from the two force plates, two additional pulling forces act on the hands of the subject. The latter forces cause the ZML to tilt, but because the subject is stable, the ZML still passes through the SP, which, in this case, extends from the subject’s hands around its back to its toes, as shown in Figure 7e. In the last frame, the subject is no longer in contact with the bench, so the external forces acting on it are the ground force plate reaction force and the pulling forces acting on its hands. The SP in this frame is very similar to the SP from the previous frame, extending from the subject’s hands around its back to its toes, which is presented in Figure 7b. Even in this scenario, the subject is stable, as shown by the ZML traversing its SP. However, if the hand contact ceased, the ZML would no longer traverse its SP. In this case, the subject would become unstable and fall on its back.

### 3.2. Two Approaches for Treating a Balanced System

**Figure 9:**
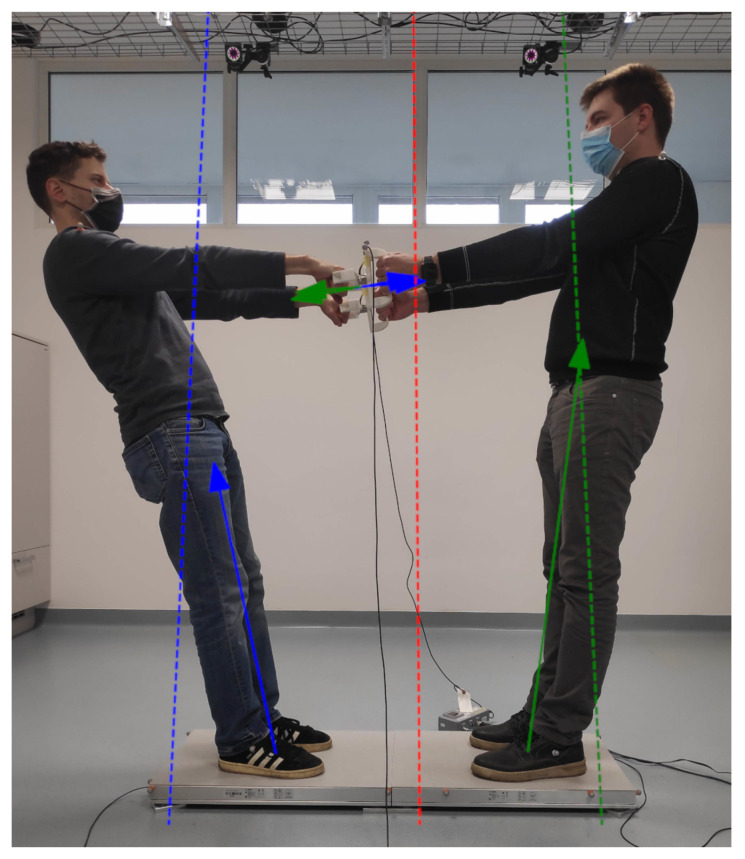
Two subjects stand on separate force plates and hold on to the double handles while leaning backward. The blue and green arrows represent the external forces acting on the subject to the left and right, respectively. The red dotted line represents the ZML of both subjects treated as one system, while the blue and green dotted lines represent the ZMLs of the subject to the left and right, respectively, when treated as separate systems.

In the second example presented in this paper, a system consisting of two subjects holding on to the double handles and leaning backward, as shown in Figure 9, is discussed. The system of two subjects can be treated in two different ways. In the first case, both subjects are treated as one system, while in the second case, each subject is treated as a separate system. In the first case, both subjects share the same SP, which extends in their sagittal plane only on the ground between their heels, while in the second case, each subject has its own SP, which extends in its sagittal plane from its hands, around its back, to its toes, as shown in Figure 7b. On the other hand, whether the two subjects are treated as one or two systems, the SP in the lateral plane extends between the outermost parts of their feet.

Because both subjects were standing still and were therefore stable, all the ZMLs obtained for each of the two cases pass through the SPs of the corresponding systems, as shown in Figure 9. The ZMLs were calculated from the external forces and torques as described in Section 2.1.2, while the measurements were performed using the force plates and force sensors presented in Section 3.1. If the subjects were to leave the grips, their respective SP would shrink to the area between their own feet and therefore both would become unstable and fall on their backs.

## 4. Discussion

The ZMP can be calculated and applied in several ways that are appropriate for particular situations. The most common way of applying this stability parameter is to express it as a location on the ground, which is described in Section 2.1.1. However, this way of expressing the ZMP gives useful results only in cases when the investigated system is supported solely on the ground and is not subjected to large horizontal accelerations. The latter can shift the location of the ZMP to such large distances from the investigated system where there are no support mechanisms and therefore stability of the system cannot be achieved. Other ways of determining system stability involve the locations, moments of inertia along with the spatial and angular accelerations of the COMs of the constituent parts of the investigated system. Furthermore, some methods are based also on the calculations of the friction cones at the contact points of the investigated system with the environment. However, some of the above-mentioned parameters may, in various situations, not be known, which means that those approaches can therefore not be applied.

To extend the use of the ZMP, we have proposed two new formulations in which the same stability criteria can be applied more generally. The first proposed solution, in this paper, is described in Section 2.1.2. Here, the stability of a system is determined by the ZML resulting from all external forces and torques. When the system is in equilibrium and Equation (7) holds, the ZML, which coincides with the COP line, passes through the SP and the force, which is the sum of all the external forces, and acts anywhere along the ZML, can balance the system. If Equation (7) does not hold, the ZML does not pass through the SP, and the sum of all the external forces cannot act on the system and make it stable. This method is particularly useful in cases where the COM of the investigated system is not known.

In the second proposed solution, presented in Section 2.1.3, the ZML was extended to the ZMP angle, defined as the angle between the ZML and the vertical line passing through the COM of the system. This method can be used when the location and acceleration of the COM of the investigated system are known. Its advantage is that it does not require the system to be stable and Equation (7) to hold, but it enables the determination of the system stability only from the measured data, i.e., the external forces, torques, their corresponding locations, and the COM of the investigated system.

The main advantage of the newly proposed methods is that they can also be used in cases when the system is not supported only on the ground but also at higher altitudes. Such scenarios can be encountered not only in humanoid robotics but in all fields of robotics, where floating-base robots interact with the environment. Furthermore, the newly proposed methods can also be applied in cases when the system is accelerated with large horizontal accelerations. Knowing the ZML or the ZMP angle, the system can be supported at such locations to create an SP that encloses the ZML or the ZMP angle. This way, support at far locations on the ground, which would be needed if the standard ground definition of the ZMP was used, can be replaced with support at different heights. On such occasions, the system can use its own support mechanisms, for example, arms in the case of a humanoid robot, or support from other external mechanisms can be applied.

The application of the ZML, obtained from the measured forces and torques and discussed in Section 3.1 in the case of a subject sitting down and standing up, shows the usefulness of this parameter. Because the subject was stable throughout the measurement, the ZML always crossed its SP. However, the ZML also shows that there are some intervals during the measurement when the subject would become unstable if the grip with the handles would be lost. Such a case is shown in the last frame of Figure 8, where the subject would become supported only by its feet and therefore the ZML would not cross the SP anymore.

In the second example given in Section 3.2, the two subjects holding on to the double handles are treated, first, as one system and then as two separate systems. In these two cases, both the SPs and the ZMLs are different. However, in each case, the ZMLs cross their corresponding SPs and reveal the stability of the treated system. This example shows that a large composed system can be treated either as a whole or each of its subsystems can be treated separately. However, in both cases, the stability parameters give the same results.

## 5. Conclusions

The stability of a robotic system is crucial when it comes to motion control because only stable systems can move in the desired way and perform the required tasks. Due to the significant progress that robotics has made in the last decades, the development of new parameters and methods that enable the motion and gait synthesis of robots is of uttermost importance for the further evolution of their capabilities.

In this paper, several ways of determining the stability parameter called the ZMP are presented. First, the standard definition is introduced where the ZMP is defined on the ground. However, this definition is only useful in situations when the system is supported solely on the ground. Second, the line definition is presented, where the COP line is calculated only from the measured external forces and torques. However, when the system is stable and the COP coincides with the ZMP, the COP line becomes the ZML, containing all possible ZMPs. The line definition is extremely useful if no other parameters of the investigated system, such as the locations and accelerations of the COMs of its constituent parts or its moments of inertia, are known. And third, the ZMP angle is defined as the angle between the ZML and a vertical line. In this case, the ZML is derived in such a way that this method can only be used if the location and the acceleration of the COM of the system under consideration, along with all the external forces and torques present, are known.

The new methods for determining stability, the ZML and the ZMP angle, defined in this paper, can be used for developing motion control systems for robots with multiple arms that do not act only on the ground. This way, the stability of humanoid robots can be evaluated in real time along with the feasibility of their desired motion. However, the application of the ZML and ZMP angle is not limited only to robots, as they can be applied to any system, including humans. One major advantage of the proposed methods is that they are not limited by the number of external forces and torques nor the locations where these forces and torques act. This makes the ZML and the ZMP angle universal parameters for determining system stability that can be applied in any situation.

## Data Availability

All the data recorded during the measurement of the subject sitting down and standing up can be provided by the authors of this paper upon request.

## Abbreviations

The following abbreviations are used in this manuscript:

ZMP	Zero moment point
ZML	Zero moment line
COM	Center of mass
SP	Support polygon

## Appendix A

Equation (17) is obtained by inserting Equations (9) and (11) in Equation (13) and considering Equation (10). Furthermore, the following relations are taken into account:

(A1) $$r_{z}^{j}\left(z_{i}\right)=s_{z}^{j}-z_{i}$$

and

(A2) $${\vec{r}}_{j}\left(z_{2}\right)-{\vec{r}}_{j}\left(z_{1}\right)=(0,0,r_{z}^{j}\left(z_{2}\right)-r_{z}^{j}\left(z_{1}\right))\;,$$

where $r_{z}^{j}\left(z_{i}\right)$ and $s_{z}^{j}$ are the *z* components of the vectors $\vec{r}\left(z_{i}\right)$ and ${\vec{s}}_{j}$, pointing from the origin of the coordinate system and the center of the plane at the height $z_{i}$, respectively, where *i* is either 1 or 2, to the point, where the *j*-th force and torque act.

(A3) $$\begin{array}{cc} k_{xz}^{\mathrm{cop}} & =\frac{z_{2}-z_{1}}{r_{x}^{\mathrm{cop}}\left(z_{2}\right)-r_{x}^{\mathrm{cop}}\left(z_{1}\right)}\\ \; & =\frac{z_{2}-z_{1}}{-\frac{M_{y}^{\mathrm{tot}}\left(z_{2}\right)}{F_{z}^{\mathrm{tot}}}+\frac{M_{y}^{\mathrm{tot}}\left(z_{1}\right)}{F_{z}^{\mathrm{tot}}}}\\ \; & =\frac{F_{z}^{\mathrm{tot}}\left(z_{2}-z_{1}\right)}{\sum_{j}{\left[{\vec{M}}_{j}+{\vec{r}}_{j}\left(z_{1}\right)\times {\vec{F}}_{j}\right]}_{y}-\sum_{j}{\left[{\vec{M}}_{j}+{\vec{r}}_{j}\left(z_{2}\right)\times {\vec{F}}_{j}\right]}_{y}}\\ \; & =\frac{F_{z}^{\mathrm{tot}}\left(z_{2}-z_{1}\right)}{\sum_{j}{\left[{\vec{F}}_{j}\times ({\vec{r}}_{j}\left(z_{2}\right)-{\vec{r}}_{j}\left(z_{1}\right))\right]}_{y}}\\ \; & =\frac{F_{z}^{\mathrm{tot}}\left(z_{2}-z_{1}\right)}{\sum_{j}[F_{x}^{j}(r_{z}^{j}\left(z_{1}\right)-r_{z}^{j}(z_{2})]}\\ \; & =\frac{F_{z}^{\mathrm{tot}}\left(z_{2}-z_{1}\right)}{\sum_{j}[F_{x}^{j}\left(s_{z}^{j}-z_{1}-s_{z}^{j}+z_{2}\right)]}\\ \; & =\frac{F_{z}^{\mathrm{tot}}\left(z_{2}-z_{1}\right)}{F_{x}^{\mathrm{tot}}\left(z_{2}-z_{1}\right)}\\ \; & =\frac{F_{z}^{\mathrm{tot}}}{F_{x}^{\mathrm{tot}}} \end{array}$$

Equation (19) is obtained by inserting Equations (9), (11) and (17) in Equation (15) and considering Equation (10). For this derivation, the plane at height $z_{1}$ was chosen.

(A4) $$\begin{array}{cc} n_{xz}^{\mathrm{cop}} & =z_{1}-k_{xz}^{\mathrm{cop}}r_{x}^{\mathrm{cop}}\left(z_{1}\right)\\ \; & =z_{1}+\frac{F_{z}^{\mathrm{tot}}}{F_{x}^{\mathrm{tot}}}\frac{M_{y}^{\mathrm{tot}}\left(z_{1}\right)}{F_{z}^{\mathrm{tot}}}\\ \; & =z_{1}+\frac{\sum_{j}{\left[{\vec{M}}_{j}+{\vec{r}}_{j}\left(z_{1}\right)\times {\vec{F}}_{j}\right]}_{y}}{F_{x}^{\mathrm{tot}}}\\ \; & =z_{1}+\frac{\sum_{j}[M_{y}^{j}+r_{z}^{j}\left(z_{1}\right)F_{x}^{j}-r_{x}^{j}\left(z_{1}\right)F_{z}^{j}]}{F_{x}^{\mathrm{tot}}}\\ \; & =z_{1}+\frac{\sum_{j}[M_{y}^{j}+(s_{z}^{j}-z_{1})F_{x}^{j}-r_{x}^{j}\left(z_{1}\right)F_{z}^{j}]}{F_{x}^{\mathrm{tot}}}\\ \; & =\frac{\sum_{j}[M_{y}^{j}+s_{z}^{j}F_{x}^{j}-r_{x}^{j}\left(z_{1}\right)F_{z}^{j}]}{F_{x}^{\mathrm{tot}}} \end{array}$$
